# EGFR enhances the stemness and progression of oral cancer through inhibiting autophagic degradation of SOX2

**DOI:** 10.1002/cam4.2772

**Published:** 2019-12-11

**Authors:** Xiao‐Xi Lv, Xiao‐Yu Zheng, Jiao‐Jiao Yu, Hua‐Rui Ma, Cheng Hua, Run‐Tao Gao

**Affiliations:** ^1^ Department of Stomatology Beijing Friendship Hospital Capital Medical University Beijing P.R. China; ^2^ Department of Pharmacology Institute of Materia Medica Chinese Academy of Medical Sciences & Peking Union Medical College Beijing P.R. China; ^3^ College of Biotechnology Tianjin University of Science & Technology Tianjin China

**Keywords:** autophagy, gefitinib, oral cancer, P62, stemness

## Abstract

Epidermal growth factor receptor (EGFR) is highly expressed in head and neck squamous cell carcinoma (HNSCC) and correlates with poor prognosis. EGFR has been demonstrated to be associated with cancer stem cell traits in HNSCC. However, the underlying molecular mechanism is far from elucidated. Here, SOX2, one of the most important stem cell markers, was identified as a binding partner and substrate of EGFR. EGFR signaling inhibition decreases SOX2 expression by promoting its autophagic degradation. Mechanistically, EGFR activation induces SOX2 phosphorylation at the Y277 site and reduces its ubiquitination, which inhibits its association with p62 and subsequent autophagic degradation. Gefitinib, an EGFR tyrosine kinase inhibitor, shows in vitro and in vivo protective effects against oral cancer cells that can be reversed through autophagy inhibition. Our study suggests that EGFR plays an important role in the development of cancer stem cells by stabilizing SOX2. Targeting EGFR in combination with conventional chemotherapy might be a promising strategy for the treatment of HNSCC through elimination of cancer stem cells.

## Graphical abstract

SOX2 is a binding partner and substrate of EGFR. EGFR activation induces SOX2 phosphorylation at Y277 site and reduces its ubiquitination, which inhibit its associated with p62 and subsequent autophagic degradation. Gefitinib, an EGFR tyrosine kinase inhibitor, decreases SOX2 expression via promoting its autophagic degradation.

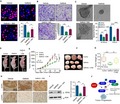

## 1 INTRODUCTION

Head and neck squamous cell carcinoma (HNSCC) has the sixth highest incidence of cancers worldwide.^1^ Despite sophisticated surgical and radiotherapeutic modalities, most HNSCC is characterized by poor prognosis and a low survival rate because of metastasis or relapse.^1^, ^2^, ^3^ A deeper understanding of the biological traits of this kind of cancer is required to improve the therapeutic efficacy against HNSCC.

Epidermal growth factor receptor (EGFR) is a receptor protein tyrosine kinase that plays an important role in regulating the survival, proliferation, and differentiation of epithelial cells as well as tumors of epithelial cell origin. High expression of EGFR occurs in more than 90% of HNSCCs.^4^, ^5^ The activation of the EGFR signaling pathway is associated with a cancer cell phenotype, inhibition of apoptosis, established angiogenesis, and increased metastatic potential.^6^ It is worth noting that a retrospective study including 47 confirmed HNSCC patients revealed that 57% of patients harbored EGFR‐TK domain mutations.^7^ EGFR regulates a specific population in HNSCC, which presents stem cell‐like properties.^8^ These facts indicated a possible link between HNSCC stemness and highly activated EGFR signaling.

Cancer stem cells (CSCs) are a distinct tumor side population that is thought to have a major role in tumor relapse and metastatic spread.^9^ Therefore, the identification of regulators that control the initiation and maintenance of HNSCC CSCs might pave the way for new therapeutic strategies to eliminate CSCs. Upregulation of stemness regulators, including c‐MYC, KLF4, SOX2, NANOG, and OCT4, has been reported to be crucial in maintaining self‐renewal properties and supporting the pluripotent state in normal somatic cells and in malignantly transformed cells by driving cellular reprogramming.^2^, ^10^ Of these, the expression of SOX2 is closely correlated with poor prognosis in HNSCC patients.^11^, ^12^, ^13^ Previous studies have reported that the expression of SOX2 is tightly controlled at both the transcriptional and protein levels.^14^, ^15^, ^16^, ^17^ However, how SOX2 is regulated in HNSCC has not been elucidated.

In this study, SOX2 was associated with EGFR. Activation of EGFR induces SOX2 phosphorylation at Tyr277, which deceases SOX2 ubiquitination and subsequent autophagic degradation. Inhibiting EGFR signaling decreases the proliferation, invasion, and tumor sphere‐forming capacity of CAL‐27 cells, a human tongue squamous cell carcinoma cell line. Moreover, the in vivo antitumor effect of gefitinib against oral cancer cells can be reversed through autophagy inhibition. Our findings suggest that EGFR signaling enhances HNSCC stemness and progression by reducing the autophagic degradation of SOX2 and increasing its stability. Gefitinib may serve as a useful combination therapeutic agent to improve the therapeutic efficacy against HNSCC via targeting CSCs.

## 2 MATERIALS AND METHODS

### 2.1 Plasmid construction

Myc‐tagged SOX2 was inserted into the pcDNA 3.1‐myc vector by standard subcloning methods. SOX2 mutants (Y277A and Y277D) were established using the Fast Mutagenesis System (TransGen Biotech). A DDK‐tagged SQSTM1 expression plasmid was purchased from Origene.

### 2.2 Cell culture

CAL‐27, a human tongue epithelial squamous cell carcinoma cell line, was purchased from the American Tissue Type Collection (ATCC). Cells were cultured in DMEM supplemented with 10% FBS, streptomycin (100 μg/mL), penicillin (100 u/mL), 0.2 mM glutamine, 0.1 mmol/L nonessential amino acids, and 1 mmol/L pyruvate and incubated in a 5% CO2 humidified atmosphere at 37°C.

### 2.3 Animal studies

Athymic BALB/c nude mice (4‐6 weeks old, male) were obtained from Vital River Lab Animal Technology Co., Ltd. and maintained in the animal facility at the Institute of Materia Medica under specific‐pathogen‐free (SPF) conditions. For animal studies, the mice were earmarked before grouping and then were randomly separated into groups by an independent experimenter, and there was no particular method for randomization. Sample size was determined according to previous experiments using the same strains and protocol. We ensured that different groups were balanced in terms of age and body weight. All animal procedures were conducted in accordance with the guidelines of ARRIVE.

### 2.4 Mouse models for tumor growth

For the generation of mouse models of tumor growth, every athymic BALB/c nude mouse received 1.5 × 10^6^ CAL‐27 cells in 100 μL PBS through subcutaneous injection into the right flank. Tumor growth was analyzed externally using Vernier calipers every day. Tumor volume (TV) was calculated by the formula Tv = W^2^ × L × 0.5.^18^ Mice were allocated to three groups, as follows: (a) saline p.o.; (b) 6.25 mg/kg/d gefitinib p.o; and (b) 6.25 mg/kg/d gefitinib p.o + 30 mg/kg/d 3‐MA i.p. Mice were treated as described from day 7 after tumor cell inoculation to sacrifice.

### 2.5 Cycloheximide treatment

To determine protein degradation, cells were incubated with the protein synthesis inhibitor cycloheximide (CHX, 10 μg/mL, Sigma‐Aldrich) for the indicated times, and the expression of the indicated proteins was evaluated by immunoblotting and quantitative analyses.

### 2.6 Bafilomycin and MG132 treatment

To determine protein degradation pathways, the cells were treated with CHX (10 μg/mL) plus either the autophagy inhibitor bafilomycin (50 nmol/L, Sigma‐Aldrich) or the UPS inhibitor MG132 (10 μmol/L, Sigma‐Aldrich) for the indicated times, and the expression of protein was determined with immunoblotting and quantitative analyses.

### 2.7 Invasion assays

To detect cell invasion, cells were starved overnight in assay media (DMEM media containing 0.4% FBS), and then single‐cell suspensions were seeded into Transwell chambers with 8‐m pore size filter membranes (5 × 10^4^ cells per well in 0.4% FBS in DMEM). Chambers were precoated with fibronectin (10 mg/mL) on the lower surface, and the polycarbonate filter was precoated with Matrigel (30 mg per well). Then, the chambers were inserted into 24‐well culture plates. Noninvaded cells on the upper side of the filter were removed with a cotton swab after being cultured for 24 hours. The invaded cells were fixed with 4% paraformaldehyde in PBS and stained with 0.5% toluidine in 2% Na_2_CO_3_. The number of invaded cells was counted using a microscope at 200× magnification in eight random fields.

### 2.8 EdU assay

CAL‐27 cells were seeded on glass coverslips. EdU was added to the culture media at a concentration of 1 μmol/L. After 24 hours, cells were fixed with 4% paraformaldehyde in PBS for 15 minutes. Then, the cells were stained by incubation for 30 minutes with 100 mmol/L Tris, 1 mmol/L CuSO_4_, 10 μmol/L fluorescent azide, and 50 mmol/L ascorbic acid. After staining, cells were washed on coverslips with TBS with 0.5% Triton X‐100 three times. Images were acquired with a confocal microscope (Olympus Microsystems).

### 2.9 qRT‐PCR

Total RNA in cells was extracted using TRIzol reagent (Invitrogen). cDNA was synthesized by reverse transcription using M‐MLV Reverse Transcriptase and Oligo (dT) Primers (Promega). The mRNA level was determined with a real‐time PCR assay. PCR amplification was performed in an 8‐tube strip format (Axygen) in triplicate. Each reaction contained 1 μL forward primer and reverse primer, 1 μL template cDNA and 1 × SYBR Green PCR Master Mix in a final volume of 20 μL and was cycled using a LineGene 9620 apparatus (Bioer). The sequences of the primers are as follows: SOX2 forward‐GGGAAATGGGAGGGGTGCAAAAGAGG; SOX2 reverse‐TTGCGTGAGTGTGGATGGGATTGGTG.

### 2.10 Immunoblot and immunoprecipitation

Cells or tissues were lysed using RIPA lysis buffer (Beyotime, P0013C). Cell lysates were resolved by SDS‐PAGE and transferred to polyvinylidene difluoride membranes (Merck Millipore, IPVH00010) for immunoblotting. Signals were detected by a Tanon 5200 chemiluminescent imaging system (Tanon). For the coimmunoprecipitation assay, total cell lysates (3 mg protein) were incubated with Protein A/G Plus‐Agarose (Santa Cruz, sc‐2003) and the indicated antibodies overnight at 4°C. After washing four times, the immunocomplex was boiled in SDS sample buffer for 5 minutes. The coprecipitates were resolved by SDS‐PAGE and subjected to immunoblot analysis. All primary antibodies used for immunoblotting in this study were purchased from Abcam. The sources of the antibodies are as follows: anti‐EPCAM (ab223582), anti‐Nanog (ab109250), anti‐OCT4 (ab181557), anti‐SOX2 (ab92494), anti‐KLF4 (ab215036), anti‐p62 (ab109012), anti‐EGFR (ab52894), anti‐phosphotyrosine (ab10321), anti‐LC3 (ab48394), anti‐Beclin1 (ab210498), and anti‐PI3KC3 (ab40776). Validation of each primary antibody for the species and application was performed according to the guidelines on the manufacturer's website.

### 2.11 Mass Spectrometry (MS) analysis

For protein identification via mass spectrometry, CAL‐27 cells were treated with EGF (100 ng/mL) for 30 minutes, and whole cell lysates were extracted and immunoprecipitated with an anti‐EGFR antibody using a Pierce CO‐IP Kit (Thermo Scientific, USA). The immunoprecipitates were resolved by SDS‐PAGE followed by silver staining; the bands in the gel were cut and subjected to LC‐MS/MS sequencing and data analysis by QLBio Biotechnology Co., Ltd. Peptides only assigned to a given protein group were considered unique.

### 2.12 Tumor sphere formation assay

CAL‐27 cells were plated in 6‐well ultralow attachment plates at a density of 1 × 10^5^ cells/well in serum‐free DMEM/F12. Fresh aliquots of human recombinant EGF (10 ng/mL) and basic fibroblast growth factor (bFGF; 20 ng/mL) were added every 2 days, and the serum‐free medium was changed every other day until sphere formation.^19^

### 2.13 Statistics

Data are represented as the mean ± standard error of the mean (SEM). Comparisons between two groups were performed using an unpaired two‐tailed Student's *t* test. Comparisons between multiple groups were performed using one‐way ANOVA with Bonferroni's multiple comparison test. Generally, all assays were carried out with n ≥ 3 biological replicates. *P* < .05 was considered statistically significant.

## 3 RESULTS

### 3.1 EGFR is highly expressed in HNSCC and interacts with SOX2

**Figure 1:**
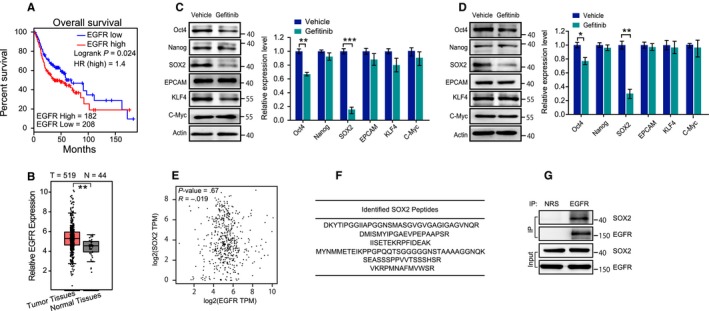
SOX2 is an interaction partner of EGFR, and its expression level is under the control of EGFR signaling. A, The association of EGFR mRNA with overall survival of HNSCC was verified in the Gene Expression Profiling Interactive Analysis (GEPIA) database. B, The mRNA expression of EGFR in human HNSCC tissues and adjacent normal tissues was analyzed using the GEPIA platform. C, CAL‐27 cells were treated with gefitinib (10 μmol/L) for 24 h; cell extracts were then prepared, and the indicated proteins were evaluated with immunoblotting. D, SCC‐15 cells were treated with gefitinib (10 μmol/L) for 24 h; cell extracts were then prepared, and the indicated proteins were evaluated with immunoblotting. E, The association between the mRNA expression of EGFR and SOX2 was verified in the GEPIA database. F, Whole cell lysates from CAL‐27 cells were immunoprecipitated with an anti‐EGFR antibody and resolved by SDS‐PAGE followed by Coomassie blue staining. The bands were extracted from the gel, and six unique peptides from SOX2 were identified by LC‐MS/MS analysis. G, Anti‐EGFR coprecipitates from CAL‐27 cells were analyzed with an anti‐SOX2 antibody to verify the interaction between EGFR and SOX2. Data are shown as representative immunoblots from three independent assays. *P < .05; **P < .01; ***P < .001

As reported, EGFR is overexpressed in over 90% of HNSCC patients, and we analyzed the relationship between EGFR expression and HNSCC survival using the GEPIA platform.^20^ A high level of EGFR is correlated with a decreased overall survival rate of patients with HNSCC (Figure 1A). In addition, higher EGFR expression was found in HNSCC tissues than in the adjacent normal tissues (Figure 1B). It has been proposed that cancer stem cells are responsible for cancer progression, the development of metastasis, and treatment failures. Although there are clues to show the possible link between the HNSCC stemness signature and highly activated EGFR signaling, the underlying molecular mechanisms are still far from elucidated. Gefitinib, a selective tyrosine kinase inhibitor of EGFR, induced a reduction in OCT4 and SOX2 levels in CAL‐27 and SCC‐15 cells, two human tongue squamous cell carcinoma cell lines. Of the two proteins, SOX2 demonstrated more significant expression changes (Figure 1C,D). However, we found that there was no correlation between EGFR and SOX2 at the mRNA level in head and neck squamous cell carcinoma (Figure 1E). Thus, the effects of gefitinib on SOX2 expression are likely due to posttranscriptional regulation. To identify potential EGFR substrates, coprecipitates of EGFR from CAL‐27 cells were sent for mass spectrometry analysis. Six unique peptides derived from SOX2 were identified in EGFR coprecipitates (Figure 1F and Table S1), suggesting SOX2 as a binding partner of EGFR. Moreover, this interaction was confirmed by coimmunoprecipitation assays (Figure 1G). These data suggest that SOX2 is an interaction partner of EGFR and that its expression level is under the control of EGFR signaling.

### 3.2 EGFR signaling increases SOX2 expression by enhancing its stability

**Figure 2:**
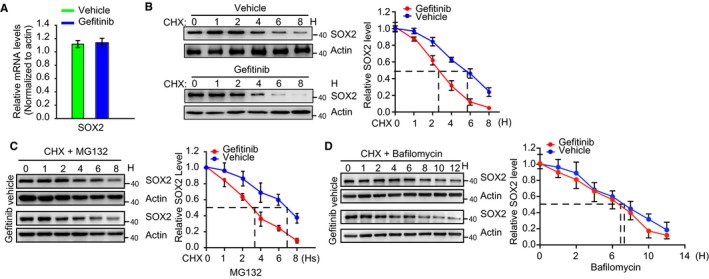
Inhibition of EGFR decreases SOX2 stability. A, CAL‐27 cells were treated with gefitinib (10 μmol/L) for 24 h. Total RNA was extracted, and the mRNA encoding SOX2 and β‐actin was detected by qPCR using specific primers. Data are presented as the mean ± SEM of three independent assays in triplicate. B, Quantitative analyses of SOX2 protein stability in gefitinib‐treated CAL‐27 cells in the presence of the protein synthesis inhibitor cycloheximide (CHX) (10 μg/mL) for the indicated times are shown (n = 3). Actin was used as a loading control for western blotting. C, Quantitative analyses of SOX2 degradation in the presence of CHX (10 μg/mL) and MG132 are shown (n = 3). D, Quantitative analyses of SOX2 degradation in the presence of CHX (10 μg/mL) and bafilomycin are shown (n = 3). Actin was used as a loading control for western blotting. Statistical significance between two groups was determined by Student's *t* test

We next sought to dissect the molecular mechanism by which EGFR increases SOX2 expression in CAL‐27 cells. Quantitative PCR analysis was carried out to determine the mRNA level of SOX2 in both vehicle‐ and gefitinib‐treated CAL‐27 cells. Gefitinib treatment showed no effect on the mRNA level of SOX2 in CAL‐27 cells (Figure 2A). Instead, gefitinib‐treated CAL‐27 cells displayed a much shorter SOX2 half‐life (~2.5 hours) than did the control cells (~5.5 hours) (Figure 2B), indicating that gefitinib treatment reduced SOX2 stability. Autophagy and the ubiquitin‐proteasome are the two major quality intracellular protein degradation systems. To determine which system was responsible for gefitinib‐induced degradation of SOX2, a CHX assay was carried out with either MG132 (an inhibitor of the proteasome) or bafilomycin (an inhibitor of the autophagy‐lysosome system). The proteasome inhibitor MG132 extended the half‐life of SOX2 in both control (~3.8 hours) and gefitinib‐treated (~7.4 hours) CAL‐27 cells without changing the SOX2 stability in either group (Figure 2C). However, inhibition of autophagy with bafilomycin reversed the effect of gefitinib on reducing SOX2 stability (Figure 2D). These data indicate that EGFR signaling inhibits autophagic degradation of SOX2 in oral cancer cells.

### 3.3 EGFR signal activation induces phosphorylation of SOX2 at Tyr277

**Figure 3:**
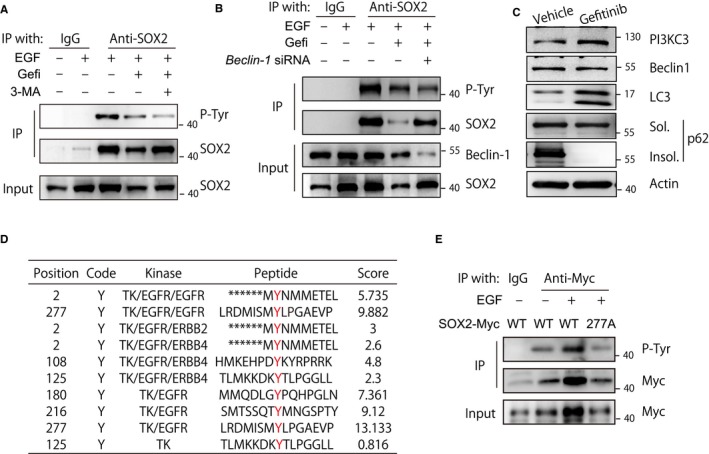
EGFR signal activation induces phosphorylation of SOX2 at Tyr277. A, CAL‐27 cells were treated with EGF (100 ng/mL) for 1 h. Before EGF stimulation, cells were pretreated with gefitinib (10 μmol/L) for 24 h with or without 3‐MA (10 μmol/L). Whole cell lysates were immunoprecipitated with an anti‐SOX2 antibody, and the indicated proteins were evaluated with immunoblotting. B, CAL‐27 cells were transfected with Beclin‐1 siRNA for 24 h and treated with EGF (100 ng/mL) for 1 h. Before EGF stimulation, cells were pretreated with gefitinib (10 μmol/L) for 24 h with or without 3‐MA (10 μmol/L). Whole cell lysates were immunoprecipitated with an anti‐SOX2 antibody, and the indicated proteins were evaluated with immunoblotting. C, CAL‐27 cells were treated with gefitinib (10 μmol/L) for 24 hours. Whole cell lysates were detected with the indicated antibodies. D, The tyrosine phosphorylation site of SOX2 was predicted using a group‐based prediction system. E, The tyrosine phosphorylation of SOX2 was detected using anti‐Myc precipitates from HEK293T cells transfected with Myc‐tagged wild‐type SOX2 or the SOX2^Y277A^ mutant

Phosphorylation is important for the regulation of protein activity and stability.^21^ To rule out the possibility that SOX2 was phosphorylated by EGFR, the CAL‐27 cell immunoprecipitates from application of anti‐SOX2 antibodies were probed with a panphosphotyrosine antibody. Under EGF treatment, tyrosine phosphorylation could be detected in SOX2 immunoprecipitates that were of a similar molecular weight as SOX2. However, this modification was prohibited by blocking the EGFR signaling pathway via gefitinib. In addition, adding 3‐MA to CAL‐27 cells together with EGF and gefitinib increased SOX2 expression levels but did not reverse gefitinib‐induced reductions in SOX2 tyrosine phosphorylation (Figure 3A). Additionally, silencing *Beclin‐1* increased the level of SOX2 in gefitinib‐treated CAL‐27 cells without enhancing the SOX2 tyrosine phosphorylation level (Figure 3B). In addition, we found that gefitinib induced autophagy in CAL‐27 cells (Figure 3C). These data indicate that SOX2 acts as a substrate of EGFR and that EGFR‐induced phosphorylation of SOX2 helps maintain SOX2 stability by preventing its autophagic degradation. Kinase prediction algorithms^22^ showed that SOX2 Tyr277 was a putative EGFR phosphorylation site (Figure 3D). To further determine whether Tyr277 was the phosphorylation site targeted by EGFR, a SOX2^Y277A^ mutant was generated. EGF treatment did not induce upregulation or tyrosine phosphorylation of the SOX2^Y277A^ mutant (Figure 3E). These data indicate that EGFR‐induced SOX2 Tyr277 phosphorylation prevents the autophagic degradation of SOX2 and enhances its stability.

### 3.4 EGFR activation reduces SOX2 ubiquitination and perturbs its association with p62

**Figure 4:**
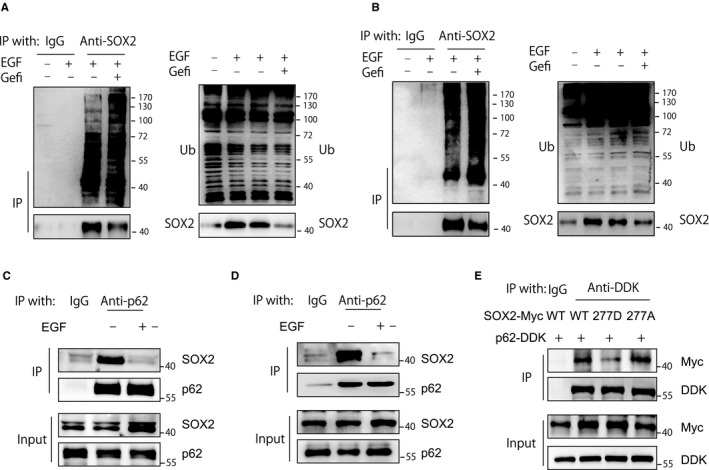
EGFR activation reduces SOX2 ubiquitination and perturbs its association with p62. A, CAL‐27 cells were stimulated with EGF (100 ng/mL) for 1 h. Before EGF stimulation, cells were pretreated with gefitinib (10 μmol/L) for 24 h. Whole cell lysates were immunoprecipitated with an anti‐SOX2 antibody, and the ubiquitination of SOX2 was evaluated with immunoblotting. B, SCC‐15 cells were stimulated with EGF (100 ng/mL) for 1 h. Before EGF stimulation, cells were pretreated with gefitinib (10 μmol/L) for 24 h. Whole cell lysates were immunoprecipitated with an anti‐SOX2 antibody, and the ubiquitination of SOX2 was evaluated with immunoblotting. C, Cell lysates from CAL‐27 cells with or without 1 hour of EGF (100 ng/mL) treatment were immunoprecipitated with an anti‐p62 antibody and blotted with the indicated antibody. D, Cell lysates from SCC‐15 cells with or without 1 hour of EGF (100 ng/ml) treatment were immunoprecipitated with an anti‐p62 antibody and blotted with the indicated antibody. E, Cell lysates from HEK293T cells transfected with the indicated plasmids were precipitated with an anti‐DDK antibody and blotted with an anti‐Myc antibody

p62 is one of the cargo receptors that mediates the degradation of ubiquitinated substrates.^23^ We found that ubiquitinated SOX2 was increased when blocking EGFR activity with gefitinib, suggesting that inhibition of EGFR activity increases SOX2 ubiquitination (Figure 4A,B). Moreover, the interaction of SOX2 with p62 was decreased after EGFR activation (Figure 4C,D). To further determine whether Y277 phosphorylation mediated the disassociation of SOX2 from p62, the interaction of p62 with wild‐type SOX2 and its Y277A and Y277D (a phosphorylation‐mimic mutant) mutants was detected. Our data showed that the Y277D mutant had a decreased binding ability with p62 in comparison with that of wild‐type SOX2 and the Y277A mutant (Figure 4E). These results demonstrate that EGFR‐induced Tyr277 phosphorylation of SOX2 reduces its binding activity with p62 and enhances its stability.

### 3.5 Autophagy inhibition reverses the antitumor effect of gefitinib

**Figure 5:**
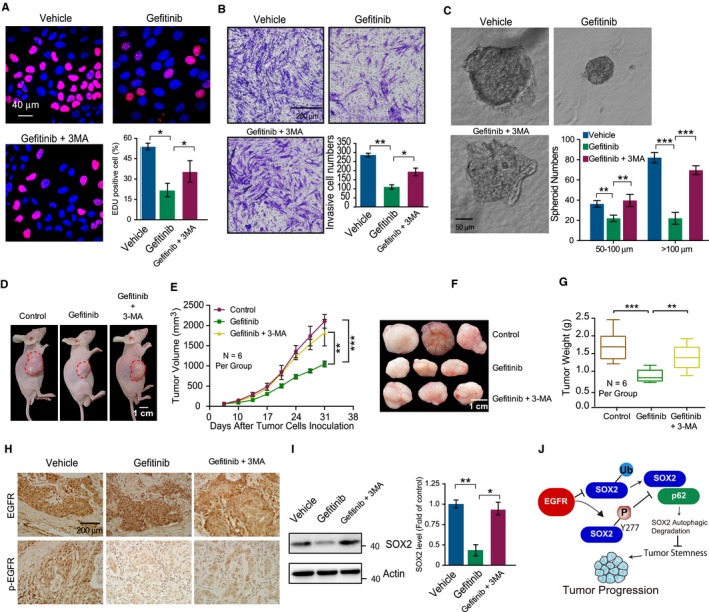
The in vitro and in vivo antitumor effects of gefitinib against CAL‐27 cells were reversed by autophagy inhibition. A, CAL‐27 cells were treated with gefitinib (10 μmol/L) for 24 h, and cell proliferation activities were evaluated by EdU labeling. The images were captured with confocal microscopy. B, Cells were treated as described in (A), and cell invasion activities were evaluated through transwell assays. C, Representative micrographs and statistical data of oncosphere growth of CAL‐27 cells treated with gefitinib (10 μmol/L) are shown. D‐G, BALB/c nude mice were s.c. inoculated with CAL‐27 cells (1.5 × 10^5^). The mice were treated with gefitinib (6.5 mg/kg/day) alone or together with 3‐MA (30 mg/kg/day) from day 7 after tumor inoculation. Data are shown from representative mice (n = 7 per group, D), as is the tumor growth curve (mean volume ± SEM) at the indicated time points (E). F, G, Tumors (F) and quantified tumor weights (G) are shown. Scale bar, 1.5 cm, n = 7 per group. H, The expression of EGFR and p‐EGFR was detected with an immunohistochemical staining assay. I, The expression of SOX2 was detected in cell lysates from CAL‐27 xenograft tumors. J, A schematic diagram illustrating the associations between EGFR and SOX2 and cancer stemness and autophagy is shown. Data are presented as the mean ± SEM. *P < .05; **P < .01; ***P < .001

Since inhibiting EGFR activity promotes autophagic degradation of SOX2, we wondered whether the tumor inhibiting effect of gefitinib could be reversed by blocking autophagy. Using the EdU cell proliferation assay, we found that the gefitinib‐decreased growth of CAL‐27 cells could be rescued to some extent by inhibiting autophagy with 3‐MA (Figure 5A). Consistently, gefitinib inhibited the invasion of CAL‐27 cells, and this could be reversed by 3‐MA treatment (Figure 5B). To identify whether gefitinib decreases the tumor‐initiating potential of oral cancer cells, we detected the sphere formation activities of CAL‐27 cells under treatment with gefitinib. Fewer oncospheres and smaller oncospheres were observed in the gefitinib treatment group than in the nontreated group, while 3‐MA treatment reversed the oncosphere‐inhibiting effect of gefitinib (Figure 5C). To investigate whether gefitinib treatment attenuates tumor growth of CAL‐27 cells in vivo, BALB/c nude mice were subcutaneously inoculated with CAL‐27 cells. One week after inoculation, the mice were treated with gefitinib (6.5 mg/kg/day) alone or in combination with 3‐MA (30 mg/kg/day). We found that control CAL‐27 cells grew at least two times faster than CAL‐27 cells with gefitinib treatment (Figure 5D‐G). However, the antitumor effect of gefitinib was reversed when mice were treated with 3‐MA simultaneously (Figure 5D‐G). In addition, gefitinib reduced the p‐EGFR level in tumor tissue, and this effect was not reversed by 3‐MA treatment (Figure 5H). However, 3‐MA abolished the effect of gefitinib on decreasing the level of SOX2 in tumor tissues (Figure 5I). Taken together, these results verify that the antitumor efficacy of gefitinib is dependent on autophagy activation and subsequent SOX2 degradation.

## 4 DISCUSSION

As with other solid tumors, HNSCC is characterized by a heterogeneous population of cancer cells with either stem cell characteristics or more differentiated traits. CSCs are closely associated with aggressive growth, metastasis, and resistance to chemotherapy or radiotherapy in HNSCC, resulting in tumor recurrence after treatment.^24^ Thus, defining the underlying mechanisms regulating the CSC traits of HNSCC may help to find targeted ways to eliminate CSCs and enhance the therapeutic effect against HNSCC. In the present study, we investigated the role and potential mechanisms of EGFR in the regulation of HNSCC stemness.

Overexpressed EGFR is one of the established hallmarks for aggressive and metastatic HNSCC.^25^, ^26^ EGFR interacts and stabilizes the stability of sodium/glucose cotransporter 1 (SGLT1) to promote the dedifferentiation and progression of oral squamous carcinoma cells.^27^, ^28^ EGFR kinase activity blockade inhibits the lymph node metastasis of oral cancer cells through selective downregulation of integrin expression and FAK phosphorylation.^29^ A previous study reported that the overexpression of EGFR supports stem cell‐like properties through upregulation of stem cell markers and promotion of tumorsphere formation.^30^ Interestingly, their data showed that the overexpression of EGFR induced the high expression of stem cell markers at the protein level but without changing gene expression, suggesting a posttranscriptional regulatory mechanism. Here, our findings provide evidence that EGFR activation induces SOX2 phosphorylation and stabilization. Activation of EGFR was reported to induce Beclin 1 phosphorylation and suppress the core autophagy machinery.^31^ On the basis of our findings, EGFR may exert a SOX2 stability‐maintaining effect through two mechanisms: (a) suppressing autophagy by inducing Beclin 1 phosphorylation and (b) destroying the association of SOX2 with p62, the autophagic cargo. These two effects together result in a defect in p62‐mediated autophagic degradation of SOX2.

The transcription factor SOX2 has been identified as one of the core regulators that maintain the self‐renewal of embryonic and cancer stem cells. The expression level of SOX2 is tightly regulated at different levels. The transcription of SOX2 can be regulated by SIRT1 in an epigenetic manner.^32^ At the protein level, SOX2 was reported to be degraded either through the ubiquitin‐proteasome system or autophagy.^17^, ^33^ SOX2 protein stability is precisely regulated through different kinds of posttranslational modifications. Ubiquitin‐conjugating enzyme E2S (Ube2s) mediates K11‐linked polyubiquitination at the Sox2‐K123 residue and facilitates proteasome‐mediated degradation.^16^ In embryonic stem cells, the precise level of SOX2 protein is regulated by a balanced methylation and phosphorylation switch.^17^ Here, we provide evidence to show that EGFR‐mediated Y277 phosphorylation is a critical modification for maintaining SOX2 stability by avoiding its autophagic degradation. Previous studies have paid some attention to the relationship between EGFR signaling and SOX2 expression. In nonsmall‐cell lung cancers, SOX2 was reported to be modulated by EGFR signaling; however, these studies only describe the phenomenon that EGFR signaling activation is connected with upregulation of SOX2 expression.^34^, ^35^ Our study not only defines how EGFR upregulates SOX2 expression but also determines the exact amino acid involved in this process.

In response to p62‐dependent autophagic clearance, the substrates are usually ubiquitinated for subsequent p62 recognition.^36^ Although the role of autophagy in cancer development is subject to debate, blocking p62 cargo function could promote cancer development by prohibiting the degradation of many oncoproteins via autophagy.^37^ In this study, we observed the induction of autophagy and SOX2 ubiquitination under gefitinib treatment. However, the exact relationship between Y277 phosphorylation and the ubiquitination of SOX2 remains to be determined. Collectively, our study provides evidence that EGFR signaling induces SOX2 phosphorylation at Y277 and decreases SOX2 ubiquitination, which inhibits the binding of SOX2 with p62 and prohibits the autophagic degradation of SOX2. These findings establish a molecular connection between activated EGFR signaling and the stem cell‐like phenotype of HNSCC (Figure 5J).

HNSCC commonly spreads to the cervical lymph nodes and results in poor outcomes. Currently, cisplatin is the first‐line therapeutic drug used for HNSCC treatment. However, over 50% of people who take cisplatin experience a recurrence of the cancer due to resistance to the drug. Cancer stem cells are the most important risk factor for tumor formation and development; these cells also self‐renew and tend to be resistant to cancer therapy. However, the optimal therapeutic strategy for treating cancer stem cells remains unclear. Here, we identified that gefitinib decreases the expression of SOX2, a critical cancer stem cell regulator, and attenuates the oncosphere formation activity of oral cancer cells. Thus, treatments using a combination of EGFR inhibitors with surgery or conventional chemotherapy will target both the bulk of the tumor and the cancer stem cell subpopulation, thereby reducing the possibility of relapse and metastasis.

## CONFLICT OF INTERESTS

The authors declare no competing financial interests.

## AUTHOR CONTRIBUTIONS

GRT raised conceptions and participated in the overall design, supervision, and coordination of the study. LXX and ZXY designed and performed most of experiments. LXX and GRT contributed to the writing, review, and revision of the manuscript. All authors read and approved the final manuscript.
